# Modeling Extinction Risk of Endemic Birds of Mainland China

**DOI:** 10.1155/2013/639635

**Published:** 2013-12-18

**Authors:** Youhua Chen

**Affiliations:** Department of Zoology, University of British Columbia, Vancouver, BC, Canada V6T 1Z4

## Abstract

The extinction risk of endemic birds of mainland China was modeled over evolutionary time. Results showed that extinction risk of endemic birds in mainland China always tended to be similar within subclades over the evolutionary time of species divergence, and the overall evolution of extinction risk of species presented a conservatism pattern, as evidenced by the disparity-through-time plot. A constant-rate evolutionary model was the best one to quantify the evolution of extinction risk of endemic birds of mainland China. Thus, there was no rate shifting pattern for the evolution of extinction risk of Chinese endemic birds over time. In a summary, extinction risk of endemic birds of mainland China is systematically quantified under the evolutionary framework in the present work.

## 1. Introduction

Global biodiversity crisis is emerging and increasingly recognized in recent years for biologists [1]. Terrestrial environment has been widely affected by humans [2] and habitats of terrestrial species are facing irreplaceable transformation which in term would pose great threats to the survival of these species. It is said that worldwide organisms are now facing the sixth mass extinction period [3, 4]. In such a context, ecologists have high pressures to facilitate conservation measures so as to better offer refuges for conserving species. One of these measures is to understand the evolution and drivers of extinction risk of species [5, 6].

Birds are an important vertebrate taxonomy and deserve to be allocated more conservation efforts because of their popularity for common people [7–9]. Understanding and modeling extinction risk of birds would be an important step to set up corresponding conservation strategies. There are growing interests focusing on the diversification, biogeography, conservation, and extinction risk of bird species [10–13].

In recent years, one of the trends in conservation biology is to sufficiently incorporate evolutionary information for the purpose to evaluate the impacts of species history on structuring species' contemporary distribution [14], conservation priorities [15, 16], or threatened risk [2, 17, 18]. One rationale for modeling extinction risk of species through phylogenetic tree is that the underlying ecological variables associated with extinction risk of species are related to evolutionary history of species, for example, distributional ranges [14, 19], morphological traits [20], physiological tolerance spectrum of environmental conditions [21], and others.

China is one of the megabiodiverse countries over the world [22]. There are many previous studies working on the systematics, ecology, and conservation of birds in China [13, 16, 23, 24]. However, understanding the extinction risk of vertebrate taxa of China from an evolutionary perspective has never been seen in any of these previous literatures. As such, in the present study, I explore the extinction risk of endemic birds of mainland China by analyzing the evolution of risk over the available species phylogenetic tree.

## 2. Materials and Methods

The list of the endemic birds of mainland China was gathered from previous studies [23, 25–27] and World Bird Database (http://avibase.bsc-eoc.org/). Threatened status of each species was collected from IUCN Red List database (http://www.iucnredlist.org/). The following categories and associated abbreviations were used: EN (endangered), VU (vulnerable), NT (near threatened), and LC (least concerned). One species *Strix davidi* did not have any records in the IUCN Red List, while another two species *Caprimulgus centralasicus *and *Leucosticte sillemi* were listed in the category of DD (data deficient). All of them were excluded for subsequent analyses. Finally, another species (*Ficedula beijingnica*) was found not to be included in the tree files of an online database described below. As such, in the present study, 48 endemic birds were included for the analyses (Table 1).

The phylogenetic relationship of these birds was extracted from the BirdTree.org database (http://www.birdtree.org/), which was derived from a full phylogeny of the global bird species in a previous study [28]. 3000 trees for the possible phylogenetic affinities of these 48 endemic birds were retrieved and the resultant consensus tree with average branch lengths was obtained using DendroPy Python library [29]. Molecular dating of the tree was fulfilled using a penalized likelihood method [30]. The resultant dated tree was used for all subsequent analyses and was available as the supplemental material of the study (see Supplementary Material available online at http://dx.doi.org/10.1155/2013/639635).

I followed some previous studies to model extinction risk of species [2, 18]. In detail, first, I assigned a discrete integer to each of the IUCN categories as follows: EN (1), VU (2), NT (3), and LC (4). Then, I applied the disparity through time (DTT) [31] to model the pattern of IUCN threatened status of species over different clades of the endemic bird phylogeny. DTT is the standardized mean pairwise distance between species [2, 31]. When the disparity of species is more remarkable between than within clades, DTT would be close to 0 towards the contemporary time and high DTT values are usually found at the time points near the root of the tree, implying that threatened status of species within a specific subclade tends to be similar. In contrast, when the disparity is more remarkable within clades, DTT should approach 1 towards the contemporary time and high DTT values are located at time points closed to the tips of the tree, implying that threatened status of species within a specific clade tends to vary greatly. For any time point, when the observed DTT value is higher than the expected one (on the randomized null curve), trait conservatism is suggested. In contrast, when the observed DTT value lies below the expected one on the null curve, trait overdispersion was suggested. As such, DTT index provided a way to understand the evolutionary paths of extinction risk of species along the evolutionary history. DTT was calculated from the extinction risk classes of endemic birds using “*geiger*” package [32] under R environment [33] with 1000-time randomization test.

I also applied different evolutionary models to model the evolution of threatened risk of species [18]. In specific, the following models were used to fit the evolution of extinction risk of species: Delta, linearChange, twoRate and null models. Detailed information about these models [18] for characterizing the temporal patterns of extinction risk of species were presented in Table 2. During the modeling, the equal-rate transition model was assumed. Model selection was performed using Akaike Information Criteria (AIC) [34]. The lower AIC the model has, the better it is.

## 3. Results

**Figure 1:**
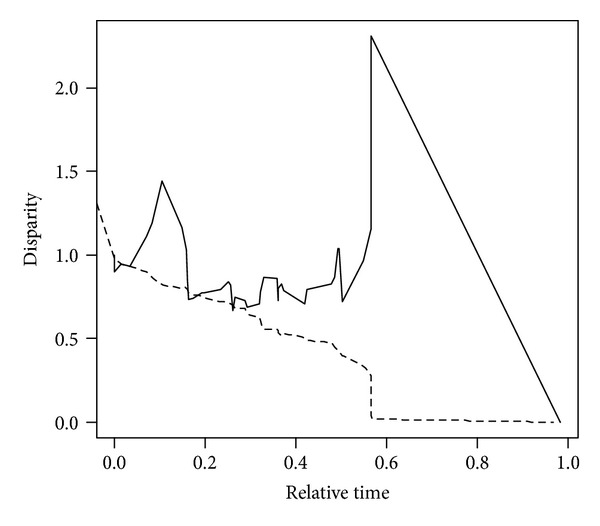
Disparity through time plot of extinction risk of endemic birds of China. The solid line is the observed curve for the endemic bird phylogeny, while the dashed line denotes the simulated curve under a 1000-randomization process. For the relative time in the *x*-axis, 0 means the root of the clade, while 1 means the present time.

As showed in Figure 1, disparity within clades above the null line indicated that extinction risk of species tends to be similar within both old and young clades (indicating phylogenetic conservatism within subclades). In particular, there was a large difference between the observed and null DTT under randomization when evolutionary time window moves towards current time (Figure 1).

Although the two-rate shifting model had the lowest AIC value (AIC = 125.46) (Table 3), it was not considerably different from the AIC value (AIC = 126.34) for the null model which assumes a single constant rate. Also, the twoRate model had a breakpoint at evolutionary time 0.013, which is almost identical to the starting point of the phylogenetic tree (hence becomes very unrealistic). Moreover, the twoRate model has one more parameter in comparison to that of the constant-rate model. Therefore, the constant-rate evolutionary rate model could not be rejected and should be retained as the best one to quantify the evolution of extinction risk of endemic birds of mainland China.

## 4. Discussion

The present short report showed that extinction risk of endemic birds of mainland China showed a conservatism pattern over evolutionary history (Figure 1). Moreover, the relative high DTT values were found at time points near the root, indicating that extinction risk of endemic birds tends to be similar as long as they are in the same subclade. At last, it was observed that the constant-rate evolutionary model is the best one to quantify the evolution of extinction risk of endemic birds of mainland China.

It was found that angiosperm and vertebrate species showed many fundamental differences at evolutionary perspectives. For example, it was found that the less threatened taxa are found in more diverse clades for vertebrates [35, 36], while the more threatened species are present in more diverse clades for plants [2]. In the present study, it was further found that vertebrates and plants can be different at the aspects of the evolutionary models for best quantifying trait evolution. A recent study working on African angiosperm species showed that Delta model was most favored [18], while in my study, the constant-rate model was the best one to explain evolution of extinction risk of endemic birds of China.

However, it shares some similarities for the evolution of extinction risk between bird and angiosperm species. For example, as mentioned above, there exists a large difference between the observed and null DTT under randomization when evolutionary time approaches current time. This implies that “late-bust” model is applicable to explain the evolution of extinction risk of bird species, being similar to that for angiosperm species in the Cape region of Africa [2].

My present study may not be generalized to the situation when taking nonendemic avian taxa into consideration. Sampling issue is very sensitive for phylogenetic comparative studies [37, 38]. As such, for future perspectives, it would be of broad implication to analyze a more comprehensive dataset by including all bird species found in mainland China so as to better quantify the evolution of threatened risk of birds.

There are a suite of limitations of the present study. First, the sampling of endemic birds of China is still incomplete. I cannot obtain either the phylogenetic positions or detailed distributional information of som other endemic bird species. Hence, they are not included in the present study, which in turn drives the conclusions of the present study to become biased more or less. The omission of nonendemic species might further lead to a bias in bias the present results, although their distributional ranges are out of the territory of China. Second, species extinction rates could be related to a variety of complicated factors, for example, the contemporary habitat conditions, climatic variability, historical contingency, and level of disturbances that the species is facing. As such, modeling of species extinction risk from an evolutionary perspective might not be of full help to elucidate the extinction mechanisms of species driven by anthropogenic disturbance.

## Supplementary Material

Phylogenetic tree reconstructed for the endemic birds of mainland China.

## Figures and Tables

**Table 1:** List of endemic bird species of China and associated IUCN categories used in the present study.

Order	Family	Species	Common name	IUCN
Galliformes	Phasianidae	*Arborophila ardens *	Hainan Partridge	VU
Galliformes	Phasianidae	*Arborophila gingica *	White-necklaced Partridge	NT
Galliformes	Phasianidae	*Arborophila rufipectus *	Sichuan Partridge	EN
Galliformes	Phasianidae	*Lophophorus lhuysii *	Chinese Monal	VU
Galliformes	Phasianidae	*Alectoris magna *	Przevalski's Partridge	LC
Galliformes	Phasianidae	*Tragopan caboti *	Cabot's Tragopan	VU
Galliformes	Phasianidae	*Syrmaticus ellioti *	Elliot's Pheasant	NT
Galliformes	Phasianidae	*Syrmaticus reevesii *	Reeves's Pheasant	VU
Galliformes	Phasianidae	*Tetraophasis obscurus *	Verreaux's Monal-Partridge	LC
Passeriformes	Certhiidae	*Certhia tianquanensis *	Sichuan Treecreeper	NT
Passeriformes	Muscicapidae	*Phoenicurus alaschanicus *	Przevalski's Redstart	NT
Passeriformes	Leiothrichidae	*Garrulax davidi *	Plain Laughingthrush	LC
Passeriformes	Leiothrichidae	*Babax koslowi *	Tibetan Babax	NT
Passeriformes	Sittidae	*Sitta yunnanensis *	Yunnan Nuthatch	NT
Passeriformes	Leiothrichidae	*Garrulax bieti *	White-speckled Laughingthrush	VU
Passeriformes	Sylviidae	*Chrysomma poecilotis *	Rufous-tailed Babbler	LC
Passeriformes	Aegithalidae	*Aegithalos fuliginosus *	Sooty Bushtit	LC
Passeriformes	Corvidae	*Perisoreus internigrans *	Sichuan Jay	VU
Passeriformes	Sylviidae	*Paradoxornis paradoxus *	Three-toed Parrotbill	LC
Passeriformes	Sylviidae	*Paradoxornis conspicillatus *	Spectacled Parrotbill	LC
Passeriformes	Sylviidae	*Paradoxornis przewalskii *	Przevalski's Parrotbill	VU
Passeriformes	Sylviidae	*Paradoxornis zappeyi *	Grey-hooded Parrotbill	VU
Passeriformes	Corvidae	*Podoces biddulphi *	Biddulph's Ground Jay	NT
Passeriformes	Leiothrichidae	*Garrulax elliotii *	Elliot's Laughingthrush	LC
Passeriformes	Cisticolidae	*Rhopophilus pekinensis *	Chinese Hill Warbler	LC
Passeriformes	Leiothrichidae	*Garrulax sukatschewi *	Snowy-cheeked Laughingthrush	VU
Passeriformes	Aegithalidae	*Leptopoecile elegans *	Crested Tit-warbler	LC
Passeriformes	Fringillidae	*Carpodacus roborowskii *	Tibetan Rosefinch	LC
Passeriformes	Urocynchramidae	*Urocynchramus pylzowi *	Przevalski's Finch	LC
Passeriformes	Fringillidae	*Carpodacus eos *	Stresemann's Rosefinch	LC
Passeriformes	Pellorneidae	*Alcippe variegaticeps *	Golden-fronted Fulvetta	VU
Passeriformes	Pellorneidae	*Alcippe striaticollis *	Chinese Fulvetta	LC
Passeriformes	Phylloscopidae	*Phylloscopus hainanus *	Hainan Leaf Warbler	VU
Passeriformes	Phylloscopidae	*Phylloscopus kansuensis *	Gansu Leaf Warbler	LC
Passeriformes	Phylloscopidae	*Phylloscopus emeiensis *	Emei Leaf Warbler	LC
Galliformes	Phasianidae	*Bonasa sewerzowi *	Chinese Grouse	NT
Passeriformes	Leiothrichidae	*Garrulax lunulatus *	Barred Laughingthrush	LC
Passeriformes	Leiothrichidae	*Garrulax maximus *	Giant Laughingthrush	LC
Passeriformes	Oriolidae	*Oriolus mellianus *	Silver Oriole	VU
Passeriformes	Paridae	*Parus davidi *	Père David's Tit	LC
Passeriformes	Emberizidae	*Emberiza koslowi *	Tibetan Bunting	NT
Passeriformes	Emberizidae	*Latoucheornis siemsseni *	Slaty Bunting	LC
Passeriformes	Leiothrichidae	*Liocichla omeiensis *	Emei Shan Liocichla	VU
Passeriformes	Paridae	*Parus superciliosus *	White-browed Tit	LC
Galliformes	Phasianidae	*Chrysolophus pictus *	Golden Pheasant	LC
Passeriformes	Paridae	*Parus venustulus *	Yellow-bellied Tit	LC
Galliformes	Phasianidae	*Crossoptilon auritum *	Blue Eared Pheasant	LC
Galliformes	Phasianidae	*Crossoptilon mantchuricum *	Brown Eared Pheasant	VU

**Table 2:** Detailed description of alternative evolutionary models used for modeling the extinction risk of endemic birds of China.

Model name	Model description
Delta	Delta < 1 describes that the evolution rate of extinction risk of species occurs rapidly early in the history of a clade and then slows through time. Delta > 1 describe an increasing evolution rate of extinction risk of species through time. Delta = 0 is identical to a Brownian motion model.

LinearChange	This model assumes that that evolution rate of extinction risk of species should change linearly overtime. If the rate is increased linearly up to the present time, then the fitting slope of the linear relationship is positive. In contrast, if the evolutionary rate is decreased linearly over the time, then the fitting slope should be negative. No change on the evolutionary rate implies that the fitting slope is zero.

TwoRate	This model allows that the evolution rate of extinction risk of species shifts to a new value at some time point over the phylogeny (if the new evolution rate is larger than 1, evolution is believed to increase, otherwise decrease). Before and after the shifting point, the evolutionary rates are kept constant.

Null	This model assumes a global constant evolutionary rate for extinction risk. Thus, only a single constant value is returned when fitting the null model.

**Table 3:** Estimated parameters of alternative evolutionary models which have been fitted onto the evolution of extinction risk for endemic birds of China.

Models	Log-likelihood	*q*	Parameters	AIC
Delta	−63.12	−1.49	0.527	128.24
LinearChange	−63.11	−0.617	2.27	128.22
TwoRate	−60.73	−0.47	*B* = 0.013, *E* = 72.20	125.46
Null	−63.17	1.09	—	126.34

*B*: breakpoint; *E*: the second rate; *q* denotes the equal transition rate among the categories of extinction risk. AIC: Akaike Information Criteria.
