# Supplementary material for: Modeling Extinction Risk of Endemic Birds of Mainland China
Source: Int J Evol Biol. 2013 Dec 18;2013:639635. doi: 10.1155/2013/639635 (PMC3878274; doi:10.1155/2013/639635)
Supplement: Supplementary file 1 — Phylogenetic tree reconstructed for the endemic birds of mainland China. [file 639635.f1.doc]

(Arborophila_rufipectus:1,Arborophila_ardens:1,Arborophila_gingica:1,(((((Urocynchramus_pylzowi:0.8262328325,((Emberiza_koslowi:0.4344859046,Latoucheornis_siemsseni:0.4344859046):0.272331254,(Carpodacus_eos:0.5062040302,Carpodacus_roborowskii:0.5062040302):0.2006131284):0.1194156739):0.01343932245,(Phoenicurus_alaschanicus:0.8034434071,(Certhia_tianquanensis:0.7353379557,Sitta_yunnanensis:0.7353379557):0.06810545135):0.0362287479):0.003131470125,((((Alcippe_variegaticeps:0.7115653278,((Paradoxornis_paradoxus:0.5801726522,Paradoxornis_conspicillatus:0.5801726522,Paradoxornis_przewalskii:0.5801726522,Paradoxornis_zappeyi:0.5801726522):0.04607957148,(Alcippe_striaticollis:0.575295799,Chrysomma_poecilotis:0.575295799,Rhopophilus_pekinensis:0.575295799):0.05095642471):0.0853131041):0.03724567606,(Liocichla_omeiensis:0.6426398452,(Garrulax_bieti:0.6391175415,Garrulax_sukatschewi:0.6391175415,Garrulax_elliotii:0.6391175415,(Garrulax_lunulatus:0.4342922782,Garrulax_maximus:0.4342922782):0.2048252634,(Garrulax_davidi:0.5142213841,Babax_koslowi:0.5142213841):0.1248961574):0.003522303681):0.1061711586):0.05869260488,((Aegithalos_fuliginosus:0.6794212534,Leptopoecile_elegans:0.6794212534):0.06374001091,(Phylloscopus_kansuensis:0.6387295027,(Phylloscopus_hainanus:0.4980343433,Phylloscopus_emeiensis:0.4980343433):0.1406951595):0.1044317616):0.0643423444):0.02953728279,(Parus_venustulus:0.5049501955,(Parus_davidi:0.4499994724,Parus_superciliosus:0.4499994724):0.05495072313):0.332090696):0.005762733554):0.008332166517,(Oriolus_mellianus:0.6803317408,(Perisoreus_internigrans:0.5216801959,Podoces_biddulphi:0.5216801959):0.1586515449):0.1708040508):0.1353204869,(Alectoris_magna:0.9676015959,((Tragopan_caboti:0.8949086539,(Lophophorus_lhuysii:0.738662381,Tetraophasis_obscurus:0.738662381):0.1562462729):0.03386807366,(Bonasa_sewerzowi:0.9179054844,((Chrysolophus_pictus:0.6703696584,(Crossoptilon_auritum:0.01605772701,Crossoptilon_mantchuricum:0.01605772701):0.6543119314):0.09630428119,(Syrmaticus_ellioti:0.6320139083,Syrmaticus_reevesii:0.6320139083):0.1346600314):0.1512315448):0.01087124314):0.03882486838):0.01885468258):0.01354372151);
